# 

**Published:** 1916-03

**Authors:** 


					﻿PUBLISHED MONTHLY.	ATLANTA	SUBSCRIPTION $1.00
JOURNAL RECORD--
OF MEDICINE 8 136]
0?; q
(Atlanta Medical and Surgical Journal, Established 1855. 7	~1
and Southern Medical Record, Established 1870.	\ v£ll(J ltd I
Vol. LX II	Atlanta, Ga , March, 1916	No. 12
				

